# Cathelicidin-related antimicrobial peptide protects against myocardial ischemia/reperfusion injury

**DOI:** 10.1186/s12916-019-1268-y

**Published:** 2019-02-20

**Authors:** Yihua Bei, Li-Long Pan, Qiulian Zhou, Cuimei Zhao, Yuan Xie, Chengfei Wu, Xiangmin Meng, Huanyu Gu, Jiahong Xu, Lei Zhou, Joost P. G. Sluijter, Saumya Das, Birgitta Agerberth, Jia Sun, Junjie Xiao

**Affiliations:** 1 Cardiac Regeneration and Ageing Lab, Institute of Cardiovascular Sciences, School of Life Science, Shanghai University, 333 Nan Chen Road, Shanghai, 200444 China; 2 School of Medicine, Jiangnan University, Wuxi, 214122 China; 3 Department of Cardiology, Tongji Hospital, Tongji University School of Medicine, Shanghai, 200065 China; 4 State Key Laboratory of Food Science and Technology, Jiangnan University, 1800 Lihu Avenue, Wuxi, 214122 Jiangsu China; 5 School of Food Science and Technology, Jiangnan University, Wuxi, 214122 China; 6 Department of Cardiology, The First Affiliated Hospital of Nanjing Medical University, Nanjing, 210029 China; 7 Department of Cardiology, Laboratory of Experimental Cardiology, University Utrecht, University Medical Center Utrecht, 3584 CX Utrecht, The Netherlands; 8 UMC Utrecht Regenerative Medicine Center, University Medical Center Utrecht, 3508 GA Utrecht, The Netherlands; 9 Cardiovascular Division of the Massachusetts General Hospital and Harvard Medical School, Boston, MA 02114 USA; 10 Department of Laboratory Medicine, Division of Clinical Microbiology, Karolinska Institutet, Karolinska University Hospital Huddinge, F68 Stockholm, Sweden

**Keywords:** Cathelicidin, CRAMP, LL-37, Ischemia/reperfusion injury, Cardiomyocyte, Apoptosis

## Abstract

**Background:**

Cathelicidins are a major group of natural antimicrobial peptides which play essential roles in regulating host defense and immunity. In addition to the antimicrobial and immunomodulatory activities, recent studies have reported the involvement of cathelicidins in cardiovascular diseases by regulating inflammatory response and microvascular dysfunction. However, the role of cathelicidins in myocardial apoptosis upon cardiac ischemia/reperfusion (I/R) injury remains largely unknown.

**Methods:**

CRAMP (cathelicidin-related antimicrobial peptide) levels were measured in the heart and serum from I/R mice and in neonatal mouse cardiomyocytes treated with oxygen glucose deprivation/reperfusion (OGDR). Human serum cathelicidin antimicrobial peptide (LL-37) levels were measured in myocardial infarction (MI) patients. The role of CRAMP in myocardial apoptosis upon I/R injury was investigated in mice injected with the CRAMP peptide and in CRAMP knockout (KO) mice, as well as in OGDR-treated cardiomyocytes.

**Results:**

We observed reduced CRAMP level in both heart and serum samples from I/R mice and in OGDR-treated cardiomyocytes, as well as reduced LL-37 level in MI patients. Knockdown of CRAMP enhanced cardiomyocyte apoptosis, and CRAMP KO mice displayed increased infarct size and myocardial apoptosis. In contrast, the CRAMP peptide reduced cardiomyocyte apoptosis and I/R injury. The CRAMP peptide inhibited cardiomyocyte apoptosis by activation of Akt and ERK1/2 and phosphorylation and nuclear export of FoxO3a. c-Jun was identified as a negative regulator of the CRAMP gene. Moreover, lower level of serum LL-37/neutrophil ratio was associated with readmission and/or death in MI patients during 1-year follow-up.

**Conclusions:**

CRAMP protects against cardiomyocyte apoptosis and cardiac I/R injury via activation of Akt and ERK and phosphorylation and nuclear export of FoxO3a. Increasing LL-37 might be a novel therapy for cardiac ischemic injury.

**Electronic supplementary material:**

The online version of this article (10.1186/s12916-019-1268-y) contains supplementary material, which is available to authorized users.

## Background

Cathelicidins (CRAMP in mouse/rat, LL-37 in human), a major group of antimicrobial peptides (AMPs), also designated as host defense peptides, serve as natural broad-spectrum antibiotics and play essential roles in regulating host defense and immunity [1, 2]. Cathelicidins are produced and/or expressed by many immune cells; epithelial cells of the intestine, airway, skin, and urinary tract; and genital cells. The immunomodulatory functions of cathelicidins have been increasingly documented in a variety of autoimmune diseases, such as psoriasis [3], systemic lupus erythematous [4], arthritis [5], atherosclerosis [6, 7], and type 1 diabetes [8]. Our previous report has shown that the gut microbiota via short-chain fatty acids could promote the production of CRAMP by pancreatic endocrine cells, protecting against autoimmune diabetes by inducing regulatory immune cells in the pancreas [8]. Actually, the non-microbicidal activities of cathelicidins, such as chemoattraction, immune cell activation, and angiogenesis, have attracted increasing attention [9, 10], and cathelicidins have also been investigated in the context of cardiovascular physiology and diseases [11].

Cathelicidins were previously identified in human atherosclerotic lesions [7], and neutrophil-derived mCRAMP (mouse cathelicidin-related antimicrobial peptide) was found to promote early atherosclerotic lesion formation in mice by enhancing monocyte recruitment [6]. Moreover, PR-39 (cathelicidin initially isolated from porcine small intestine) could reduce NADPH oxidase activity [12], leukocyte recruitment and adherence [13–16], and endothelial dysfunction [17], and thus may protect against ischemic and hypoxic injury [13–16]. In addition to inflammatory response and microvascular injury, the apoptosis of cardiomyocytes is a critical pathological process during cardiac ischemia/reperfusion (I/R) injury. However, the role and underlying mechanisms of cathelicidins in myocardial apoptosis upon I/R injury remain largely unclear.

In the present study, we observed reduced level of the mCRAMP peptide in both heart and serum samples from cardiac I/R mice, as well as in an oxygen glucose deprivation/reperfusion (OGDR)-induced apoptosis model of cardiomyocytes. Also, we detected decreased level of the human cathelicidin LL-37 peptide in serum samples from patients with acute myocardial infarction (MI). These data indicate a potential correlation between CRAMP and myocardial apoptosis during I/R injury. Here, we report data about the function of CRAMP in regulating cardiomyocyte apoptosis and I/R injury in vitro and in vivo and present a protective effect of CRAMP in reducing myocardial apoptosis during I/R injury by activating the protein kinase B (Akt) and extracellular signal-regulated kinases (ERK1/2) pathways, and the phosphorylation and nuclear export of forkhead box O3a (FoxO3a).

## Methods

### Myocardial infarction patients

All human investigations conformed to the principles outlined in the Declaration of Helsinki and were approved by the institutional review committees of Tongji Hospital (2014-002). The MI patients and healthy controls were recruited with a written informed consent at Tongji Hospital (Shanghai, China) from July 2015 to June 2017. Venous blood was collected at enrollment, and the serum level of LL-37 was measured by ELISA (CUSABIO).

### Animals and CRAMP knockout mice

All animal experiments were conducted under the guidelines on the use and care of laboratory animals for biomedical research published by the National Institutes of Health (No. 85-23, revised 1996), and approved by the committee on the Ethics of Animal Experiments of Shanghai University. Male C57BL/6 mice aged 8 to 10 weeks old were purchased from Cavens Lab Animal (Changzhou, China) and maintained in a specific pathogen-free (SPF) laboratory animal facility of Shanghai University (Shanghai, China). The mouse CRAMP (mCRAMP) knockout (CRAMP-KO) mice were maintained and bred as previously reported [8, 18]. To determine the cardiac phenotype of CRAMP-KO mice, the heart weight, heart weight/body weight ratio, heart weight/tibia length ratio were examined. The hematoxylin-eosin staining was used to examine cardiac structure. The wheat germ agglutinin (WGA) staining (1:50, Sigma) was performed to measure cardiomyocyte area using frozen heart sections.

### Cardiac ischemia-reperfusion injury model

Cardiac I/R injury was induced by ligation of the left anterior descending artery (LAD) for 30 min followed by cardiac reperfusion for 24 h as previously reported [19, 20]. To study the role of CRAMP in I/R injury, mice were intraperitoneally injected with the mCRAMP peptide (4 mg/kg/day, 0.5 mg/mL diluted in PBS) or vehicle control (PBS) for three consecutive days as previously reported [8], and then subjected to I/R injury or sham operation on the last day of CRAMP injection. The mCRAMP peptide used in the present study was bought from Innovagen AB (Lund, Sweden). Moreover, CRAMP global knockout (KO) mice and age-matched wild type (WT) C57BL/6 mice were subjected to I/R injury or sham operation. Twenty-four hours after reperfusion, 1 mL of 1% Evans blue was slowly injected into the left ventricle and the hearts were stained with 2,3,5-triphenyltetrazolium chloride (TTC) as reported previously [19]. The area at risk/left ventricle weight (AAR/LV) ratio and the infarct size/area at risk (INF/AAR) ratio were determined to evaluate the homogeneity of surgery and the severity of cardiac I/R injury, respectively.

### Primary cardiomyocyte isolation, culture, and treatment

Neonatal rat cardiomyocytes (NRCMs) were isolated from 1- to 3-day-old Sprague-Dawley (SD) rats and cultured in Dulbecco’s modified Eagle’s medium (DMEM; Corning) containing 4.5 g/L glucose supplemented with 10% horse serum (Gibco) and 5% fetal bovine serum (FBS, BioInd, Israel) as described before [20, 21]. To induce apoptosis, cardiomyocytes were treated with oxygen glucose deprivation/reperfusion (OGDR). Briefly, cardiomyocytes were first cultured for 8 h with serum-free no glucose DMEM (Gibco) in an air-tight chamber with a humidified hypoxic atmosphere containing 5% CO_2_ and 95% N_2_ at 37 °C. After exposure to oxygen glucose deprivation for 8 h, the culture medium was replaced with serum and glucose-containing DMEM and transferred to a normal incubator for recovery for 12 h.

To study the role of CRAMP in OGDR-induced apoptosis, NRCMs were simultaneously treated with the rat CRAMP (rCRAMP) bought from Innovagen AB (Lund, Sweden) at a dose of 0.1 mg/L for 48 h, or transfected with the siRNA targeting rCRAMP (100 nM, Ribobio, Guangzhou) for 48 h. To study whether protein kinase B (Akt) and extracellular signal-regulated kinases (ERK1/2) activation contributed to the role of CRAMP in OGDR-induced apoptosis, NRCMs were treated with the rCRAMP (0.1 mg/L, 48 h) in the presence or absence of Akt inhibitor MK2206 (10 nM, 24 h, Selleck) or MEK inhibitor PD98059 (50 μM, 24 h, Selleck). To clarify the upstream regulators of CRAMP, NRCMs were transfected with c-Jun or Rela siRNAs (100 nM, Ribobio, Guangzhou) in the presence or absence of rCRAMP siRNA for 48 h. In all in vitro experiments, the OGDR-induced apoptosis (8 h deprivation/12 h reperfusion) was conducted in the last 20 h of cell treatment.

### Immunofluorescent and TUNEL staining

Terminal deoxynucleotidyl transferase-mediated dUTP in situ nick end labeling (TUNEL) staining was conducted to detect apoptotic nuclei by confocal microscopy in α-actinin-labeled cardiomyocytes as described before [21]. Briefly, neonatal rat cardiomyocytes (NRCMs) or frozen mouse heart sections were fixed with 4% paraformaldehyde (PFA), permeabilized with 0.5% Triton X-100 in PBS, and blocked with 5% bovine serum album (BSA) before incubation with mouse anti-α-actinin (Sigma, A7811, 1:200 dilution). After incubated with Cy3-AffiniPure goat anti-mouse IgG (H+L) (Jackson), cells or tissue sections were stained with TUNEL FITC Apoptosis Detection Kit (Vazyme) according to the manufacturer’s instructions. Nuclei were counterstained with DAPI. Finally, 20–30 fields per sample were viewed under a confocal microscope (Carl Zeiss). The percentage of TUNEL-positive cardiomyocytes was calculated to determine apoptosis induced by OGDR or I/R injury.

### The levels of mCRAMP and LL-37 peptides measured by ELISA

Mouse heart tissues were harvested after I/R injury (30 min of ischemia and 24 h of reperfusion). Neonatal mouse cardiomyocytes (NMCMs) and neonatal mouse cardiac fibroblasts (NMCFs) were isolated from 1- to 3-day-old C57BL/6 mice as described before [21, 22]. NMCMs were treated with OGDR for induction of apoptosis as described above. Mouse heart tissues and cardiac myocytes and fibroblasts were rinsed and homogenized with PBS, submitted to alternate freezing and thawing, and centrifuged at 5000×*g* for 5 min at 4 °C. Mouse serum samples were collected from angular vein and centrifuged at 1000×*g* for 15 min at 4 °C. Human serum samples were obtained by percutaneous cubital venipuncture drawn in serum collecting tubes and centrifuged at 1000×*g* for 15 min at 4 °C. Supernatants from mouse samples were taken for measurement of the level of mCRAMP peptide using the mouse CRAMP ELISA kit (CUSABIO, CSB-E15061m). Supernatants from human samples were taken for measurement of the serum level of LL-37 using the human cathelicidin antimicrobial peptide (LL-37) ELISA kit (CUSABIO, CSB-EL004476HU) according to the manufacturer’s instructions.

### Western blotting

Heart tissues or cardiomyocytes were lysed with RIPA lysis buffer (Beyotime, China) complemented with 1% phenylmethylsulfonyl fluoride (PMSF) and Pierce™ protease and phosphatase inhibitor (Thermo, 88668). Equal quantities of total proteins were separated in 10% SDS-PAGE gels, transferred onto PVDF membranes, and blocked with 5% BSA. Proteins were blotted with primary antibodies at 4 °C overnight as follows: rabbit-anti-Bax (Abclonal, A0207), rabbit-anti-Bcl-2 (Abclonal, A2845), rabbit-anti-Caspase-3 (Cell Signaling, 9662), mouse-anti-pAkt (473) (Cell Signaling, 4051), rabbit-anti-pAkt (308) (Cell Signaling, 2965), rabbit-anti-Akt1 (Proteintech, 10176-2-AP), rabbit-anti-pERK1/2 (Abclonal, AP0472), rabbit-anti-ERK1/2 (Abclonal, A0229), rabbit-anti-c-Jun (Abclonal, A0246), rabbit-anti-Rela (Abclonal, A2711), rabbit-anti-VDR (Abclonal, A2194), and rabbit-anti-C/EBPα (Proteintech, 18311-1-AP). The blots were then incubated with the corresponding secondary antibodies, and protein bands were visualized using enhanced chemiluminescence (ECL) kit in ChemiDoc XRS Plus luminescent image analyzer (Bio-Rad). The β-actin (Bioworld, BS13278) and GAPDH (Bioworld, AP0063) were used as loading controls. To study the nuclear export of FoxO3a, the nuclear and cytoplasmic total proteins were separately extracted from NRCMs using Nuclear and Cytoplasmic Protein Extraction Kit (Keygen Biotech, Jiangsu, China). Equal quantities of nuclear or cytoplasmic proteins were subjected to Western blotting for rabbit-anti-pFoxO3a (S253) (Cell Signaling, 9466), rabbit-anti-pFoxO3a (T32) (Cell Signaling, 9464), and rabbit-anti-FoxO3a (Abclonal, A0102) as described above. The β-actin (Bioworld, BS13278) and Histone3H3 (Abclonal, A2348) were used as loading controls for cytoplasmic and nuclear proteins, respectively. All membranes were probed, stripped, and then reprobed for determining the phosphorylation levels of Akt, ERK1/2, and FoxO3a.

### Quantitative real-time polymerase chain reaction (qRT-PCR)

Total RNAs in NRCMs were extracted using Trizol reagent (TaKaRa) and cDNAs were synthesized using iScript™ cDNA Synthesis Kit (Bio-Rad). qRT-PCR was performed using Takara SYBR Premix Ex Taq™ (Tli RNaseH Plus, Japan) on Roche LightCycler480 PCR System. 18s or GAPDH were used as internal controls. Sequences for qRT-PCR primers are shown as follows: mouse *cTnT* forward: 5′-TCTGCCAACTACCGAGCCTAT-3′, reverse: 5′-CTCTTCTGCCTCTCGTTCCAT-3′; mouse *cTnI* forward: 5′-CAGAGGAGGCCAACGTAGAAG-3′, reverse: 5′-CTCCATCGGGGATCTTGGGT-3′; mouse *Col1a1* forward: 5′-GTAACTTCGTGCCTAGCAACA-3′, reverse: 5′-CCTTTGTCAGAATACTGAGCAGC-3′; mouse *Col3a1* forward: 5′-GGAGCACCTGGACTAGACG-3′, reverse: 5′-GCCTTGGACTGGTAAGCCAT-3′; mouse *ANP* forward: 5′-AGCCGTTCGAGAACTTGTCTT-3′, reverse: 5′-CAGGTTATTGCCACTTAGGTTCA-3′; mouse *BNP* forward: 5′-GAGGTCACTCCTATCCTCTGG-3′, reverse: 5′-GCCATTTCCTCCGACTTTTCTC-3′; rat *CRAMP* forward: 5′-TGTAGCAAGGCATCACAGCA-3′, reverse: 5′-CTTTTCGGAGGAGTCCAGCC-3′; rat *c-Jun* forward: 5′-CGTTCCGGATGGCACTCTG-3′, reverse: 5′-GAGGTCGTTGAATCTCGCCA-3′; rat *Rela* forward: 5′-TCACCAAAGACCCACCTC-3′, reverse: 5′-AGGGGTTATTGTTGGTCT-3′; rat *GAPDH* forward: 5′-ACAGCAACAGGGTGGTGGAC-3′, reverse: 5′-TTTGAGGGTGCAGCGAACTT-3′; and mouse/rat *18s* forward: 5′-TGCGGAAGGATCATTAACGGA-3′, reverse: 5′-AGTAGGAGAGGAGCGAGCGACC-3′.

### Statistical analysis

All experimental data were analyzed using SPSS (version 20.0) and presented as mean ± SD using GraphPad Prism 7.0 unless otherwise stated. An independent-sample *t* test was used for comparison between two groups. One-way ANOVA followed by Bonferroni’s post hoc test was used for comparison among more than three groups. Comparisons for clinical characteristics between two groups of human subjects were performed using the independent-sample *t* test. Binary logistic regression analysis was performed to examine the association of the serum level of LL-37 with clinical features. Univariate and multivariate analyses were conducted to determine the independent predictors of readmission and/or death in MI patients. A receiver-operator characteristic (ROC) curve was used to assess the sensitivity and specificity of serum level of LL-37/neutrophil ratio in prediction of worse prognosis in MI patients. *P* values < 0.05 were considered statistically significant.

## Results

### The CRAMP peptide is reduced upon cardiac I/R injury

**Fig. 1:**
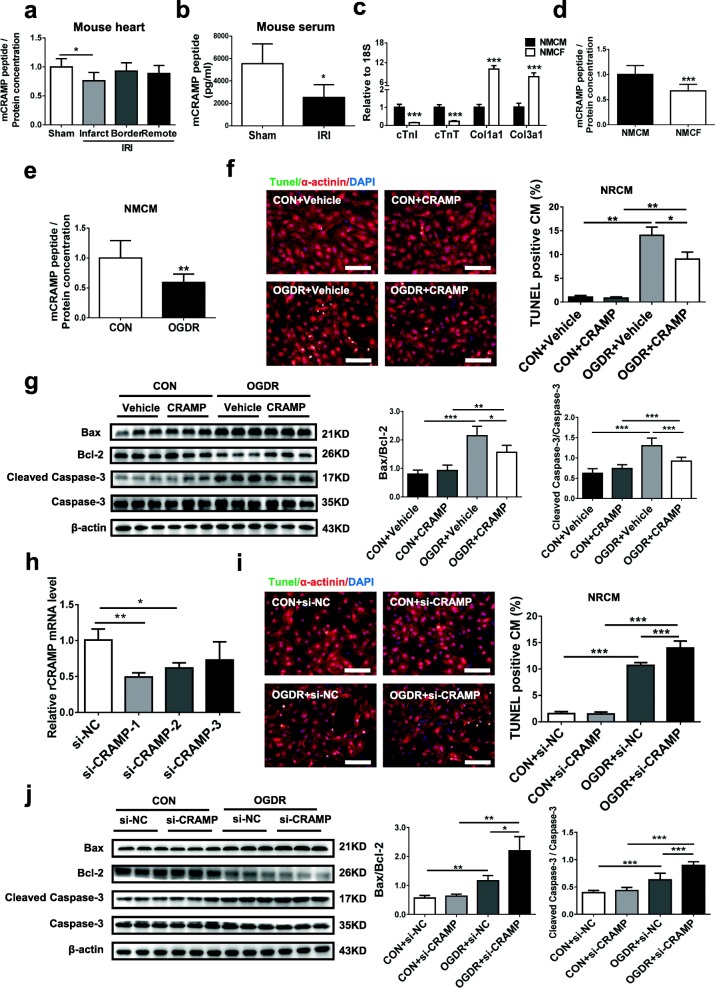
CRAMP is reduced in cardiac ischemia/reperfusion (I/R) injury and prevents cardiomyocyte apoptosis. **a** The level of the mCRAMP peptide was measured by ELISA in the infarct, border, and remote zones of mouse I/R hearts compared to a sham group (*n* = 5). **b** The level of the mCRAMP peptide was measured by ELISA in the serum from I/R mice compared to a sham group (*n* = 5). **c** qRT-PCRs were performed to measure specific genes expressed in isolated neonatal mouse cardiac myocytes (NMCMs) and fibroblasts (NMCFs) (*n* = 6). **d** The level of the mCRAMP peptide was measured by ELISA in NMCMs and NMCFs (*n* = 9). **e** The level of the mCRAMP peptide was measured by ELISA in NMCMs treated with oxygen glucose deprivation/reperfusion (OGDR) (*n* = 9). **f**, **g** The ratio of apoptosis after rCRAMP stimulation in OGDR-treated neonatal rat cardiomyocytes (NRCMs) as determined by TUNEL staining (**f**, *n* = 4) and Western blot (**g**, *n* = 6). Immunofluorescent staining for α-actinin was used to label cardiomyocytes. **h** The level of rCRAMP mRNA in NRCMs after transfection with siRNAs targeting rCRAMP (*n* = 3). **i**, **j** The ratio of apoptosis after transfection with rCRAMP siRNA in OGDR-treated NRCMs as determined by TUNEL staining (**i**, *n* = 4) and Western blot (**j**, *n* = 6). Immunofluorescent staining for α-actinin was used to label cardiomyocytes. Scale bar = 100 μm (**f**, **i**). Data were expressed as mean ± SD. *P < 0.05; **P < 0.01; ***P < 0.001

Using a murine model of cardiac I/R injury, we first evaluated the level of the mCRAMP peptide by ELISA in both serum and heart samples. After 30 min of cardiac ischemia and 24 h of reperfusion, the level of the mCRAMP peptide was significantly reduced in the infarct zone (Fig. 1a) as well as in the serum (Fig. 1b) compared with sham-operated mice, suggesting a role for CRAMP in I/R injury. As multiple cell types are present in the heart, we further isolated neonatal mouse cardiac myocytes (NMCMs) and fibroblasts (NMCFs) and determined the level of mCRAMP peptide in different cell types in the heart. NMCMs expressed high levels of cTnT and cTnI, while NMCFs predominantly expressed Col1a1 and Col3a1 (Fig. 1c). As measured by ELISA, we found that cardiomyocytes expressed higher level of the mCRAMP peptide compared to fibroblasts (Fig. 1d), possibly suggesting a more prominent role of CRAMP in cardiomyocytes. Moreover, we measured the cellular level of the mCRAMP peptide in NMCMs treated with oxygen glucose deprivation/reperfusion (OGDR), a cell model to mimic I/R injury and cardiomyocyte apoptosis in vitro. The level of the mCRAMP peptide was reduced in OGDR-treated cardiomyocytes (Fig. 1e). Collectively, these data show a strong correlation between CRAMP and myocardial I/R injury. We therefore sought to determine the possible role of CRAMP in processes associated with I/R injury such as cardiomyocyte apoptosis.

### CRAMP prevents cardiomyocyte apoptosis

Based on the OGDR-induced apoptosis model in neonatal rat cardiomyocytes (NRCMs), we observed that the addition of the rat CRAMP (rCRAMP) peptide led to a reduction of cardiomyocyte apoptosis as determined by TUNEL staining (Fig. 1f) and Western blot (Fig. 1g). To determine the effect of loss-of-function for CRAMP, siRNAs targeting rCRAMP were transfected to NRCMs, among which si-CRAMP (sequence 1) was the most efficient to reduce the CRAMP mRNA level and was therefore used in subsequent experiments (Fig. 1h). Knockdown of CRAMP significantly aggravated OGDR-induced apoptosis as determined by TUNEL staining (Fig. 1i) and Western blot (Fig. 1j). Taken together, these data indicate that CRAMP is protective against cardiomyocyte apoptosis.

### CRAMP activates Akt and ERK1/2 pathways

**Fig. 2:**
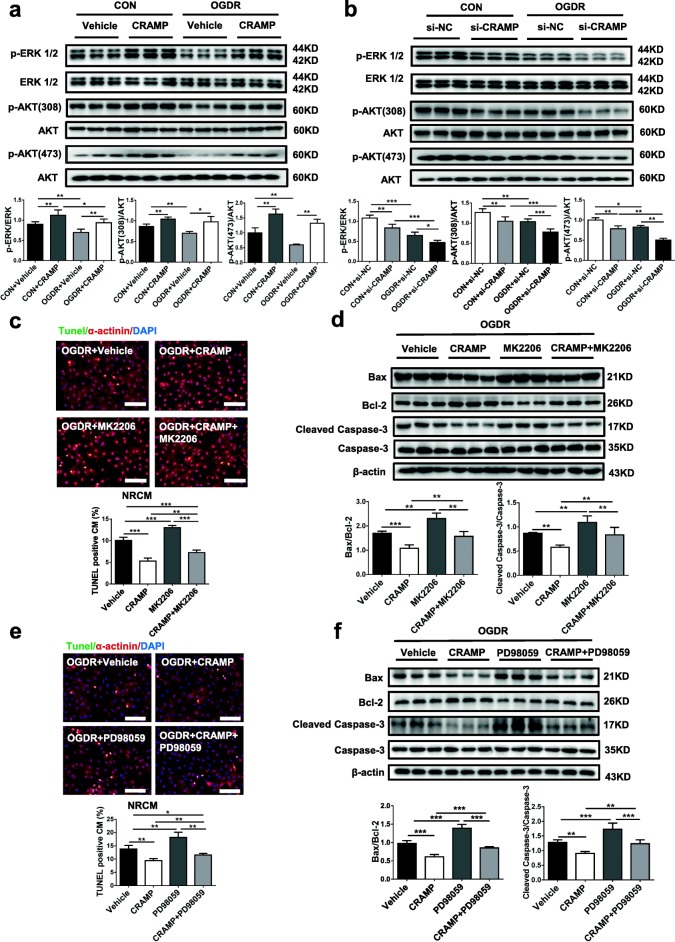
CRAMP reduces cardiomyocyte apoptosis via activating Akt and ERK1/2. **a**, **b** Western blot analysis for Akt and ERK1/2 phosphorylation after treatment of neonatal rat cardiomyocytes (NRCMs) with the rCRAMP peptide (**a**, *n* = 6) or the siRNA targeting rCRAMP (**b**, *n* = 6), in the presence or absence of oxygen glucose deprivation/reperfusion (OGDR) treatment. All membranes were probed, stripped, and then reprobed for determining the phosphorylation levels of Akt and ERK1/2. **c**–**f** The apoptosis of OGDR-treated NRCMs after treatment with the Akt inhibitor MK2206 or the MEK inhibitor PD98059 together with the rCRAMP peptide as determined by TUNEL staining (**c**, **e**, *n* = 4) and Western blot (**d**, **f**, *n* = 6). Immunofluorescent staining for α-actinin was used to label cardiomyocytes. Scale bar = 100 μm (**c**, **e**). Data were expressed as mean ± SD. *P < 0.05; **P < 0.01; ***P < 0.001

The activation of Akt and ERK1/2 pathways has been well-established as protective against myocardial injury and cardiomyocyte apoptosis [19, 23]. By Western blot, we observed that the levels of phospho-Akt and phospho-ERK1/2 were decreased in OGDR-treated NRCMs, which were rescued by adding the rCRAMP peptide (Fig. 2a). In contrast, knockdown of rCRAMP could further reduce the phospho-Akt and phospho-ERK1/2 levels in OGDR-treated NRCMs (Fig. 2b). These data implicate the Akt and ERK1/2 pathways in the role of CRAMP in cardiomyocyte apoptosis. To investigate whether Akt and ERK1/2 phosphorylation is necessary for the protective effect of CRAMP in cardiomyocytes, we treated OGDR-induced apoptotic cardiomyocytes with the rCRAMP peptide in the presence of Akt inhibitor MK2206 (10 nM) or MEK inhibitor PD98059 (50 μM). MK2206 is a specific inhibitor of Akt [24, 25], and PD98059 inhibits ERK1/2 signaling at the level of MEK [19, 26]. Here, Western blot showed that MK2206 significantly reduced Akt phosphorylation level and PD98059 reduced ERK1/2 phosphorylation level in OGDR-treated NRCMs, regardless of treatment with the rCRAMP peptide (Additional file 1: Figure S1). Using TUNEL staining and Western blot, we found that inhibiting Akt and MEK enhanced OGDR-induced cardiomyocyte apoptosis and could partially abolish the protective effect of CRAMP against cardiomyocyte apoptosis (Fig. 2c–f). These data reveal that the activation of Akt and ERK1/2 pathways is involved in the protective effect of CRAMP in reducing cardiomyocyte apoptosis.

### CRAMP increases FoxO3a phosphorylation and nuclear export

**Fig. 3:**
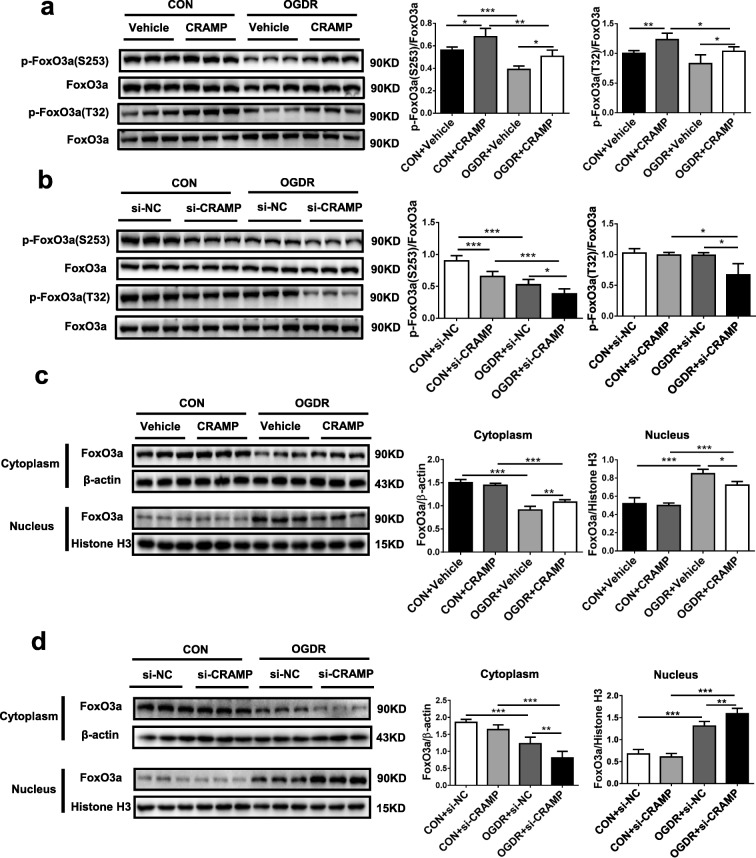
CRAMP increases FoxO3a phosphorylation and nuclear export in apoptotic cardiomyocytes. **a**, **b** Western blot analysis for FoxO3a phosphorylation after stimulation with the rCRAMP peptide (**a**, *n* = 6) or transfection with the siRNA targeting rCRAMP (**b**, *n* = 6) in neonatal rat cardiomyocytes (NRCMs) treated with oxygen glucose deprivation/reperfusion (OGDR) or not. All membranes were probed, stripped, and then reprobed for determining the phosphorylation level of FoxO3a. **c**, **d** Western blot analysis for FoxO3a subcellular expression in the cytoplasm or nucleus after stimulation with the rCRAMP peptide (**c**, *n* = 6) or transfection with the siRNA targeting rCRAMP (**d**, *n* = 6) in NRCMs treated with OGDR or not. Data were expressed as mean ± SD. *P < 0.05; **P < 0.01; ***P < 0.001

Forkhead box O3a (FoxO3a) is a nuclear forkhead transcription factor that mediates cell apoptosis by transcription of pro-apoptotic genes [27]. Previous studies have indicated that Akt and ERK1/2 can induce FoxO3a phosphorylation and subsequent nuclear export, thereby leading to reduced cell apoptosis [28]. To examine whether FoxO3a phosphorylation is increased by treatment of NRCMs with the rCRAMP peptide, contributing to its anti-apoptotic effect, we measured FoxO3a phosphorylation and subcellular location in OGDR-treated NRCMs in the presence of the rCRAMP peptide. By Western blot, we observed that the rCRAMP peptide was sufficient to increase FoxO3a phosphorylation in cardiomyocytes regardless of OGDR treatment (Fig. 3a). In contrast, knockdown of rCRAMP reduced FoxO3a phosphorylation (Fig. 3b). OGDR treatment resulted in a nuclear accumulation of FoxO3a and decreased expression of FoxO3a in the cytoplasm (Fig. 3c, d). In OGDR-treated NRCMs, the rCRAMP peptide led to FoxO3a nuclear export (Fig. 3c), while knockdown of rCRAMP further increased its nuclear accumulation (Fig. 3d). These data suggest that CRAMP can increase FoxO3a phosphorylation and nuclear export in OGDR-treated cardiomyocytes, which may be the mechanistic underpinning of the cardioprotective effect of CRAMP against cardiomyocyte apoptosis.

### The CRAMP peptide reduces cardiac I/R injury in vivo

**Fig. 4:**
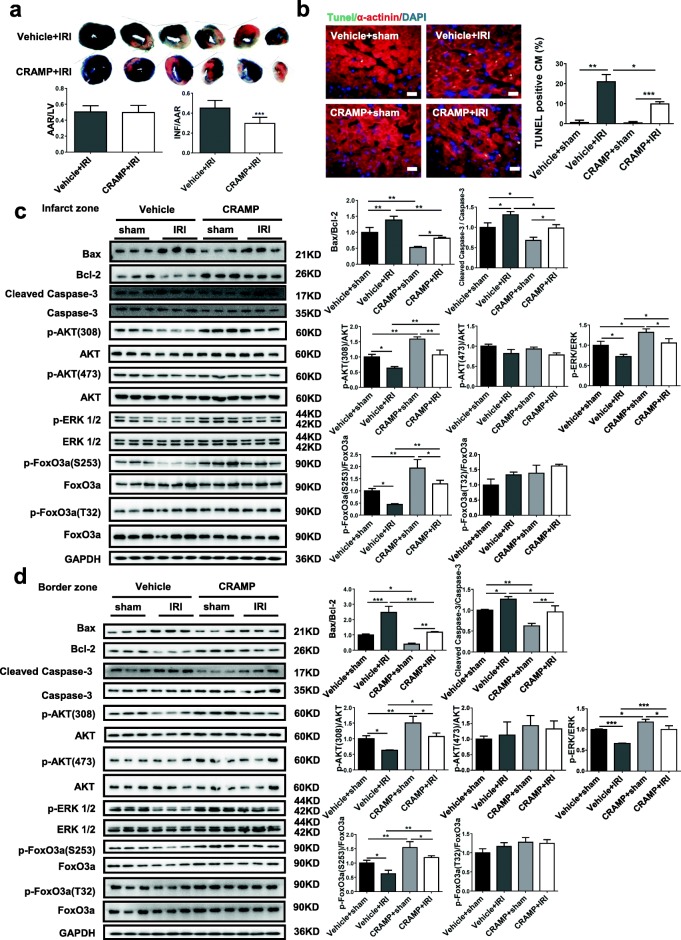
CRAMP reduces cardiac I/R injury in vivo. **a**, **b** Mice were intraperitoneally injected with the mCRAMP peptide, and the infarct size and myocardial apoptosis after I/R injury were analyzed by 2,3,5-triphenyltetrazolium chloride (TTC) staining (**a**, *n* = 11) and TUNEL staining (**b**, *n* = 4). Immunofluorescent staining for α-actinin was used to label cardiomyocytes. **c**, **d** The ratio of Bax/Bcl-2 and Caspase-3 cleavage, in addition to the Akt, ERK1/2, and FoxO3a phosphorylation levels, were analyzed by Western blot after mCRAMP treatment in both the infarct (**c**) and border (**d**) zones of mice I/R hearts (*n* = 3). All membranes were probed, stripped, and then reprobed for determining the phosphorylation levels of Akt, ERK1/2, and FoxO3a. Scale bar = 20 μm (**b**). Data were expressed as mean ± SD. *P < 0.05; **P < 0.01; ***P < 0.001

To evaluate whether CRAMP is protective against cardiac I/R injury in vivo, mice were intraperitoneally injected with the mCRAMP peptide (4 mg/kg/day) for 3 consecutive days and then subjected to I/R surgery. We first confirmed that treatment with the mCRAMP peptide significantly increased the level of the mCRAMP peptide in both serum and heart samples from I/R mice (Additional file 2: Figure S2). By TTC staining, we observed a significant decrease in the infarct size in I/R mice treated with the mCRAMP peptide (Fig. 4a). Also, TUNEL staining showed that myocardial apoptosis was reduced by mCRAMP treatment in I/R mice (Fig. 4b). These data provide direct evidence for the in vivo protective effect of CRAMP against cardiac I/R injury and myocardial apoptosis. Next, we measured apoptosis-associated proteins, as well as Akt, ERK1/2, and FoxO3a phosphorylation in cardiac tissues. We first confirmed that Bax/Bcl-2 ratio and Caspase-3 cleavage were both increased in the infarct and border areas of I/R heart, whereas the phosphorylation levels of Akt, ERK1/2, and FoxO3a were reduced (Additional file 3: Figure S3). Furthermore, CRAMP could reduce Bax/Bcl-2 ratio and Caspase-3 cleavage and activate Akt, ERK1/2, and FoxO3a phosphorylation in both sham and I/R hearts (Fig. 4c, d). These data provide in vivo evidence that the CRAMP peptide probably confers protection against I/R injury via Akt and ERK1/2 activation and FoxO3a phosphorylation.

### Deletion of the CRAMP gene exacerbates cardiac I/R injury in vivo

**Fig. 5:**
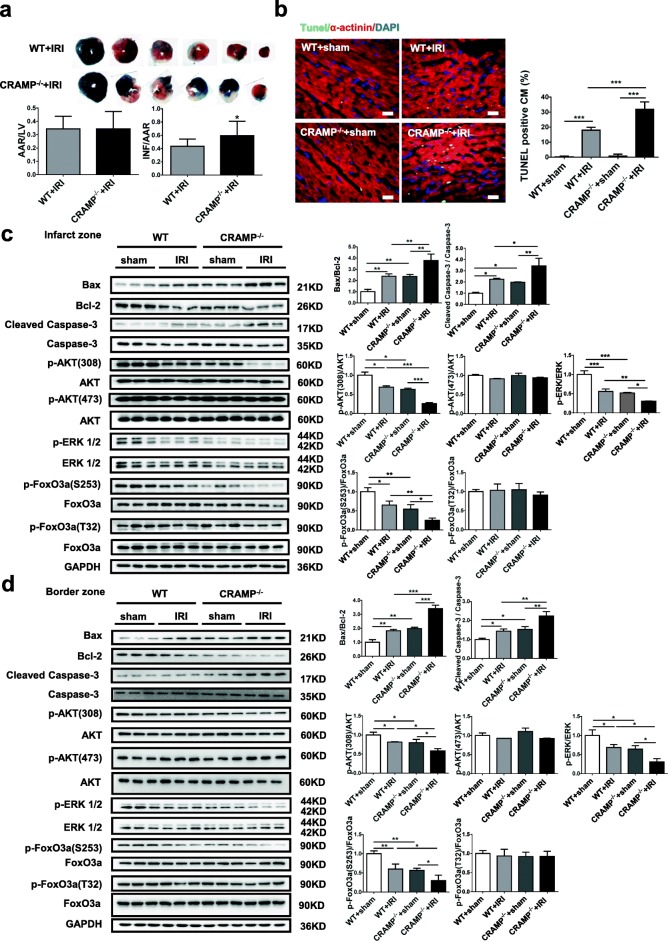
CRAMP knockout mice display increased cardiac I/R injury. **a**, **b** The infarct size and myocardial apoptosis were measured upon I/R injury of CRAMP^−/−^ mice as determined by 2,3,5-triphenyltetrazolium chloride (TTC) staining (**a**, *n* = 10) and TUNEL staining (**b**, *n* = 4). Immunofluorescent staining for α-actinin was used to label cardiomyocytes. **c**, **d** The ratio of Bax/Bcl and Caspase-3 cleavage, in addition to the Akt, ERK1/2, and FoxO3a phosphorylation levels, were analyzed by Western blot in both the infarct (**c**) and border (**d**) zones of CRAMP^−/−^ mice I/R hearts (*n* = 3). All membranes were probed, stripped, and then reprobed for determining the phosphorylation levels of Akt, ERK1/2, and FoxO3a. Scale bar = 20 μm (**b**). Data were expressed as mean ± SD. *P < 0.05; **P < 0.01; ***P < 0.001

To examine whether CRAMP deficiency would exacerbate cardiac I/R injury, mCRAMP global knockout mice were utilized. The mCRAMP knockout mice were previously reported to be normally fertile and have no obvious phenotypes as compared to WT mice [18]. Moreover, our data showed no difference in the heart weight, cardiac structure, cardiomyocyte area, and ANP and BNP expression levels between mCRAMP knockout mice and WT mice (Additional file 4: Figure S4). However, mCRAMP knockout significantly increased infarct size and myocardial apoptosis after I/R injury, as determined by TTC staining (Fig. 5a) and TUNEL staining (Fig. 5b). Moreover, Bax/Bcl-2 ratio and Caspase-3 cleavage were further increased, whereas Akt, ERK1/2, and FoxO3a phosphorylation levels were decreased in both infarct and border areas of I/R hearts (Fig. 5c, d). These data suggest that CRAMP deficiency causes increased myocardial apoptosis after I/R injury, which may be related to the inactivation of Akt and ERK1/2 pathways and inhibition of FoxO3a phosphorylation.

### c-Jun is a negative regulator of CRAMP

**Fig. 6:**
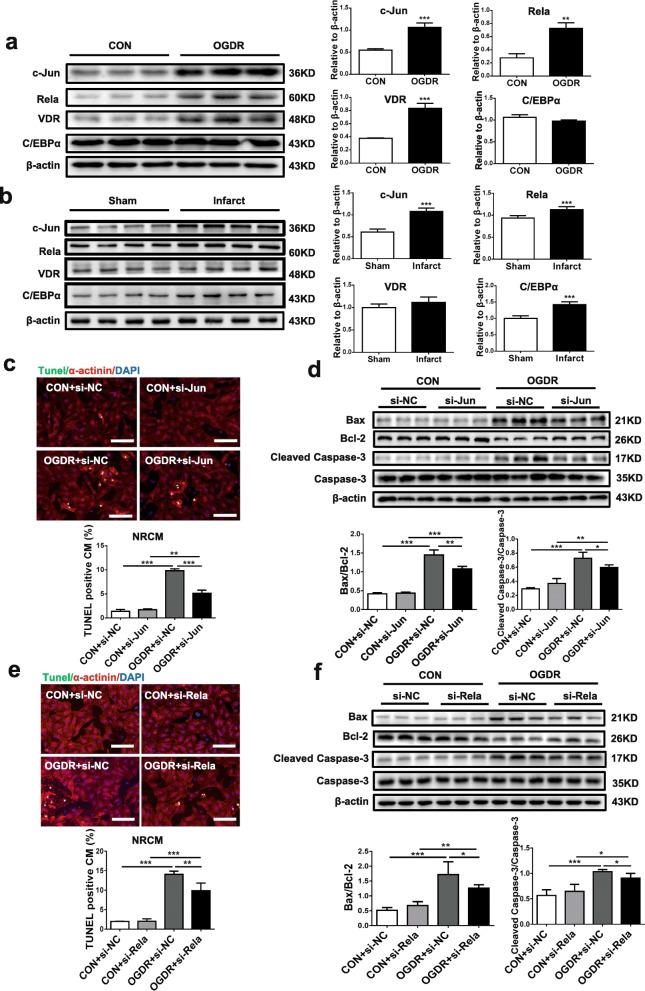
Identification of potential regulators of CRAMP and their effect in cardiomyocyte apoptosis. **a** The protein levels of c-Jun, Rela, VDR, and C/EBPα were analyzed by Western blot in neonatal rat cardiomyocytes (NRCMs) treated with oxygen glucose deprivation/reperfusion (OGDR) (*n* = 3). **b** The protein levels of c-Jun, Rela, VDR, and C/EBPα were analyzed by Western blot in heart tissues from I/R mice (*n* = 4). **c**–**f** The effects of c-Jun siRNA and Rela siRNA in OGDR-treated NRCMs as determined by TUNEL staining (**c**, **e**, *n* = 4) and Western blot (**d**, **f**, *n* = 6). Immunofluorescent staining for α-actinin was used to label cardiomyocytes. Scale bar = 100 μm (**c**, **e**). Data were expressed as mean ± SD. *P < 0.05; **P < 0.01; ***P < 0.001

**Fig. 7:**
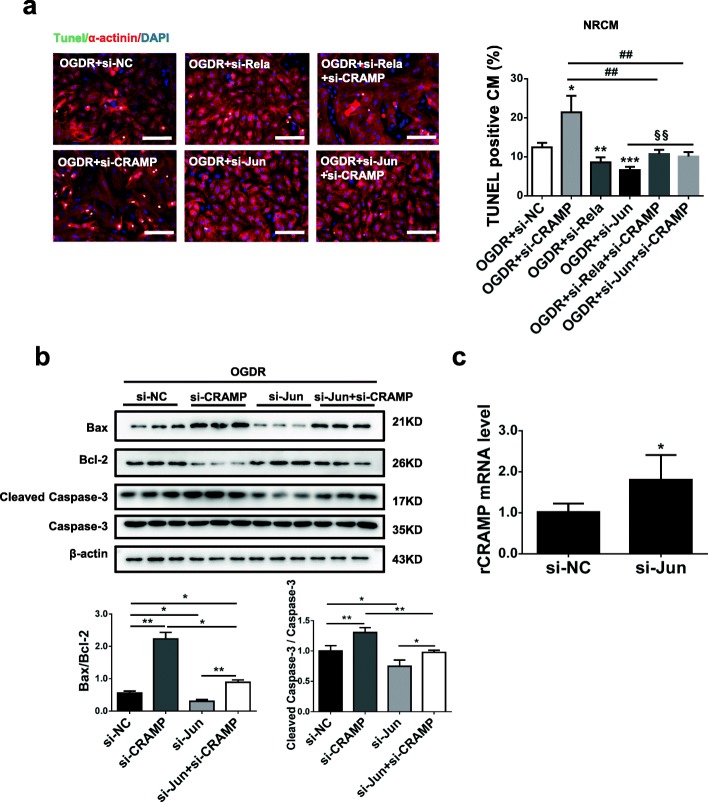
c-Jun negatively regulates CRAMP in the control of cardiomyocyte apoptosis. **a** The cardiomyocyte apoptosis was measured by TUNEL staining after transfection with siRNAs targeting Rela, c-Jun, and rCRAMP in neonatal rat cardiomyocytes (NRCMs) treated with oxygen glucose deprivation/reperfusion (OGDR) (*n* = 4). Immunofluorescent staining for α-actinin was used to label cardiomyocytes. **b** The cardiomyocyte apoptosis was measured by Western blot (*n* = 3) after transfection with siRNAs targeting c-Jun and rCRAMP in NRCMs treated with OGDR. **c** qRT-PCR for rCRAMP mRNA level in NRCMs transfected with c-Jun siRNA (*n* = 4). Scale bar = 100 μm (**a**). Data were expressed as mean ± SD. *P < 0.05 vs. OGDR+si-NC group; **P < 0.01 vs. OGDR+si-NC group; ***P < 0.001 vs. OGDR+si-NC group; ^##^*P* < 0.01 vs. OGDR+si-CRAMP group; ^§§^*P* < 0.01 vs. OGDR+si-Jun group

To further investigate the potential upstream regulators of CRAMP, we applied Genomatix Software Suite [29], which predicted c-Jun, Rela, VDR, and C/EBPα as potential regulatory candidates. By Western blot, we observed that c-Jun and Rela were both upregulated in OGDR-treated NRCMs as well as in the infarct area of mouse I/R hearts (Fig. 6a, b). Next, transfections of siRNA targeting c-Jun or Rela were performed in NRCMs (Additional file 5: Figure S5) to evaluate their effects in OGDR-induced apoptosis. Both c-Jun and Rela siRNAs were able to reduce OGDR-induced NRCM apoptosis as evidenced by TUNEL staining and Western blot (Fig. 6c–f). Function-rescue assays were further performed in OGDR-treated NRCMs using co-transfection of rCRAMP siRNA and siRNAs targeting c-Jun or Rela. Knockdown of rCRAMP was not sufficient to attenuate the protective effect of Rela siRNA against OGDR-induced apoptosis (Fig. 7a), while knockdown of rCRAMP abolished the effect of c-Jun siRNA in reducing OGDR-induced apoptosis, as evidenced by TUNEL staining (Fig. 7a) and Western blot (Fig. 7b). Finally, we validated that the rCRAMP mRNA level was significantly upregulated in NRCMs transfected with c-Jun siRNA (Fig. 7c). Collectively, these data indicate that c-Jun negatively regulates the CRAMP gene in the control of myocardial apoptosis.

### The serum level of LL-37 is reduced in patients with myocardial infarction

**Fig. 8:**
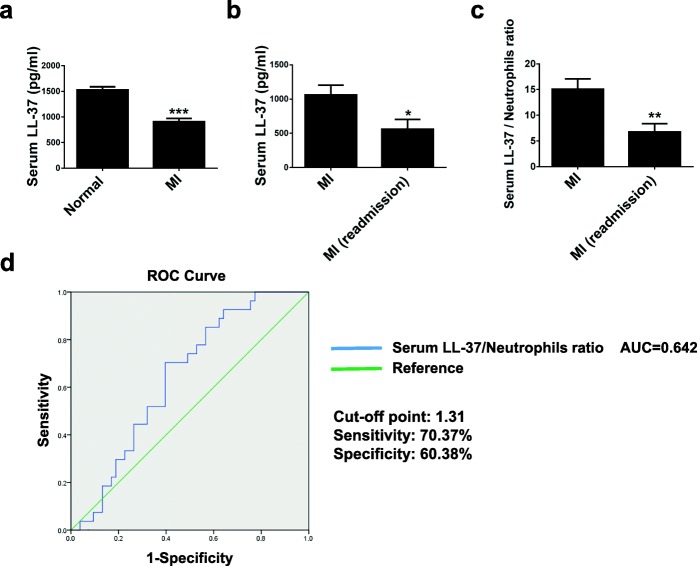
The serum level of LL-37 is reduced in patients with myocardial infarction. **a** The serum level of the human cathelicidin LL-37 was measured by ELISA in patients with myocardial infarction (MI, *n* = 172) and normal controls (*n* = 160). **b** The serum level of LL-37 was measured by ELISA in MI patients with cardiovascular readmission and/or death (*n* = 27) compared to those without readmission and/or death (*n* = 53) during the 1-year follow-up. **c** The serum LL-37/neutrophil ratio was determined in MI patients with cardiovascular readmission and/or death (*n* = 27) compared to those without readmission and/or death (*n* = 53) during the 1-year follow-up. **d** The receiver-operator characteristic (ROC) curve was used to assess the sensitivity and specificity of the serum LL-37/neutrophil ratio in prediction of readmission and/or death in MI patients. Data were expressed as mean ± SEM. *P < 0.05; **P < 0.01; ***P < 0.001

To evaluate the clinical relevance of the human cathelicidin peptide LL-37 (human analogue of CRAMP), the serum levels of LL-37 were measured using ELISA in patients with myocardial infarction (MI, *n* = 172) compared to normal controls (*n* = 160). It was found that LL-37 serum levels were significantly reduced in MI patients (Fig. 8a). Then, male MI patients with 1-year follow-up were divided into two subgroups: MI with cardiovascular readmission and/or death (*n* = 27) versus MI without cardiovascular readmission and/or death (*n* = 53). The clinical characteristics of these patients were listed in Table 1, which demonstrated that creatine kinase-MB (CK-MB, ng/mL) and neutrophils (%) were significantly higher in the readmission/death MI group compared to the no-readmission/death MI group. On the contrary, LL-37 serum levels were more reduced in MI patients with readmission and/or death (Fig. 8b). It was previously reported that MI was associated with decreased level of LL-37 and increased level of human neutrophil peptide-1 to 3 (HNP1–3, also known as α-defensins which represent the most abundant proteins in human neutrophils) in the systemic circulation [30, 31]. We therefore determined serum LL-37/neutrophil ratio in MI patients with readmission and/or death. Our data showed that serum LL-37/neutrophil ratio was significantly lower in MI patients with readmission and/or death (Fig. 8c), suggesting that serum LL-37/neutrophil ratio might be associated with worse prognosis in MI patients.

**Table 1:** Clinical characteristics of male patients with myocardial infarction (no. or mean ± SEM)

	Survival (*n* = 53)	Readmission/death (*n* = 27)	*P* value
Age (years)	59.02 ± 1.79	63.07 ± 3.19	0.234
Male sex (no.)	53	27	
Weight (kg)	74.15 ± 1.44	72.24 ± 2.52	0.481
BMI	25.60 ± 0.45	24.94 ± 0.69	0.414
Heart rate (beats/min)	80 ± 2	82 ± 4	0.550
Systolic blood pressure (mmHg)	127.79 ± 3.35	130.15 ± 3.67	0.663
Diastolic blood pressure (mmHg)	76.92 ± 1.75	73.78 ± 2.14	0.280
Killips (no.)			
I	42	16	
II	6	5	
III	1	1	
IV	4	5	
Troponin I (ng/mL)	45.25 ± 4.67	58.80 ± 5.08	0.054
Myoglobin (ng/mL)	505.19 ± 106.32	743.96 ± 183.25	0.232
CK-MB (ng/mL)	145.60 ± 15.39	206.06 ± 18.16	0.019
INR	1.04 ± 0.04	1.07 ± 0.02	0.650
D-dimer (mg/L)	0.70 ± 0.23	1.03 ± 0.31	0.400
Neutrophils (%)	77.78 ± 1.48	80.35 ± 1.61	0.002
C-reactive protein (mg/L)	18.34 ± 3.71	31.86 ± 9.89	0.209

*BMI* body mass index, *CK-MB* creatine kinase isoenzyme, *INR* international normalized ratio

To examine whether LL-37 serum level was associated with clinical features, male MI patients were further divided into two groups based on the mean value of LL-37 serum levels: LL-37 high-level group (*n* = 35) versus LL-37 low-level group (*n* = 45). The independent-sample *t* test showed that CK-MB serum level was significantly higher in LL-37 low-level group (Table 2), whereas the binary logistic regression indicated that LL-37 serum level was not associated with these clinical features of MI patients (Table 3).

**Table 2:** Clinical characteristics of male patients with myocardial infarction in groups with different serum levels of LL-37 (no. or mean ± SEM)

	LL-37 low level (*n* = 45)	LL-37 high level (*n* = 35)	*P* value
Age (years)	61.20 ± 2.38	59.34 ± 2.05	0.569
Male sex (no.)	45	35	
Weight (kg)	72.66 ± 1.69	74.60 ± 1.95	0.452
BMI	25.19 ± 0.52	25.61 ± 0.54	0.584
Heart rate (beats/min)	81 ± 2	80 ± 3	0.750
Systolic blood pressure (mmHg)	132.96 ± 3.35	122.97 ± 3.70	0.050
Diastolic blood pressure (mmHg)	77.24 ± 1.73	74.09 ± 2.20	0.255
Killips (no.)			
I	32	26	
II	7	4	
III	2	0	
IV	4	5	
Troponin I (ng/mL)	54.98 ± 4.28	43.19 ± 5.99	0.104
Myoglobin (ng/mL)	680.64 ± 133.15	463.80 ± 128.73	0.255
CK-MB (ng/mL)	200.60 ± 14.79	121.52 ± 18.19	0.001
INR	1.07 ± 0.04	1.03 ± 0.03	0.413
D-dimer (mg/L)	0.84 ± 0.28	0.78 ± 0.23	0.867
Neutrophils (%)	77.19 ± 1.27	72.95 ± 2.11	0.091
C-reactive protein (mg/L)	19.76 ± 4.17	26.95 ± 7.90	0.424

*BMI* body mass index, *CK-MB* creatine kinase isoenzyme, *INR* international normalized ratio

**Table 3:** Association of serum LL-37 level with clinical features of male patients with myocardial infarction

Variable	Odds ratio	95% CI	*P* value
Age	0.991	0.960–1.022	0.564
Weight	1.015	0.976–1.056	0.450
BMI	1.038	0.909–1.185	0.580
Heart rate	0.996	0.969–1.023	0.747
Systolic blood pressure	0.980	0.959–1.000	0.054
Diastolic blood pressure	0.979	0.943–1.016	0.253
Killips	1.033	0.661–1.614	0.887
Troponin I	0.988	0.974–1.002	0.104
Myoglobin	1.000	0.999–1.000	0.261
CK-MB	0.993	0.988–1.000	0.261
INR	0.360	0.028–4.623	0.433
D-dimer	0.976	0.739–1.289	0.865
C-reactive protein	1.005	0.993–1.017	0.395
LL-37/neutrophils	2.558	0.975–6.708	0.056

*BMI* body mass index, *CK-MB* creatine kinase isoenzyme, *INR* international normalized ratio

To further explore the predictors for MI readmission and/or death, the univariate logistic regression analysis was first performed, which identified CK-MB and serum LL-37/neutrophil ratio as two independent variables related to MI readmission and/or death (Table 4). Next, CK-MB and serum LL-37/neutrophil ratio were further subjected to multivariate logistic regression analysis, which indicated that lower level of serum LL-37/neutrophil ratio correlated with higher risk of MI readmission and/or death (OR 0.942; 95% CI 0.899–0.987; *P* = 0.012) (Table 5). Finally, the receiver-operator characteristic (ROC) curve analysis showed that the area under curve (AUC) for serum LL-37/neutrophil ratio was 0.642 (Fig. 8d). Using a cutoff point of 1.31, serum LL-37/neutrophil ratio predicted readmission and/or death with a sensitivity of 70.37% and a specificity of 60.38% in MI patients (Fig. 8d). Collectively, our results suggest that lower serum LL-37/neutrophil ratio might be predictive for worse prognosis in MI patients.

**Table 4:** Univariate analysis for predictors of readmission and/or death in myocardial infarction patients

Variable	Odds ratio	95% CI	*P* value
Age	1.020	0.987–1.055	0.233
Weight	0.984	0.942–1.028	0.478
BMI	0.940	0.811–1.089	0.411
Heart rate	1.009	0.981–1.038	0.546
Systolic blood pressure	1.005	0.984–1.026	0.658
Diastolic blood pressure	0.979	0.941–1.018	0.277
Killips	1.515	0.957–2.400	0.076
Troponin I	1.014	0.999–1.029	0.077
Myoglobin	1.000	1.000–1.001	0.238
CK-MB	1.005	1.001–1.010	0.022
INR	1.612	0.204–12.741	0.651
D-dimer	1.124	0.847–1.492	0.417
C-reactive protein	1.009	0.997–1.022	0.139
LL-37/neutrophils	0.942	0.899–0.987	0.012

*BMI* body mass index, *CK-MB* creatine kinase isoenzyme, *INR* international normalized ratio

**Table 5:** Multivariate analysis for predictors of readmission and/or death in myocardial infarction patients

Variable	Odds ratio	95% CI	*P* value
LL-37/neutrophils	0.942	0.899–0.987	0.012

## Discussion

**Fig. 9:**
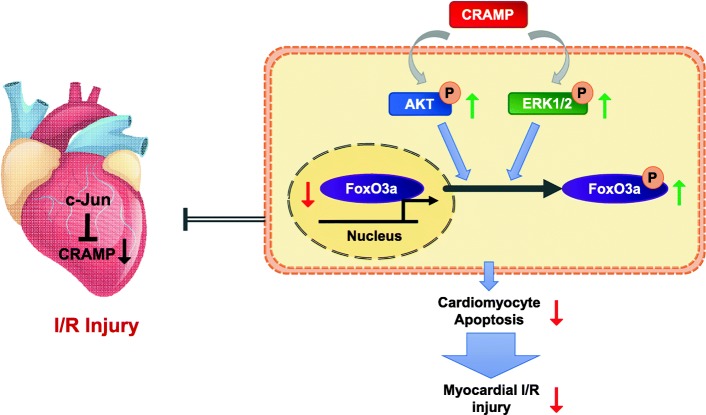
The proposed mechanism for the protective effect of CRAMP against cardiomyocyte apoptosis and myocardial ischemia/reperfusion (I/R) injury. Induced c-Jun and reduced CRAMP were identified in the heart upon I/R injury. CRAMP protects against cardiomyocyte apoptosis and myocardial I/R injury via the activation of Akt and ERK1/2 pathways and the phosphorylation and nuclear export of FoxO3a

In this study, we for the first time provide evidence for the non-inflammation regulatory and immunomodulatory function of CRAMP in protecting against myocardial I/R injury by inhibiting cardiomyocyte apoptosis. We observed decreased level of the CRAMP peptide in both heart and serum samples from I/R mice in vivo, and in OGDR-induced apoptotic cardiomyocytes in vitro. Interestingly, treatment with the CRAMP peptide was sufficient to inhibit OGDR-induced cardiomyocyte apoptosis and reduce infarct size and myocardial apoptosis upon I/R injury in mice. In contrast, knockdown of CRAMP enhanced OGDR-induced cardiomyocyte apoptosis, and CRAMP knockout mice displayed increased I/R injury. We further demonstrated that these effects were mediated by the activation of Akt and ERK1/2 pathways and the phosphorylation and nuclear export of FoxO3a. In addition, c-Jun was identified as an upstream negative regulator of the CRAMP gene. The proposed pathways are shown in Fig. 9. Interestingly, we also observed that the serum LL-37 level was decreased in MI patients and lower serum LL-37/neutrophil ratio might be predictive of worse prognosis for MI patients.

Myocardial I/R injury is triggered by a series of pathophysiological processes, including oxidative stress, inflammation, intracellular calcium overload, cardiomyocyte apoptosis, and impaired angiogenesis [32, 33]. Increasing studies have implicated the role of cathelicidins in cardiovascular physiology and diseases [11]. PR-39, a porcine cathelicidin, was identified as a powerful inhibitor of phagocyte NADPH oxidase [12] and has been reported to reduce ischemic and hypoxic injury by regulating inflammatory response and microvascular dysfunction [13–17]. However, the role of cathelicidins in modulating myocardial apoptosis upon I/R injury remained largely unknown.

In the present study, we first demonstrated that the level of the CRAMP peptide was decreased in the infarct area of I/R hearts as well as in the apoptotic cardiomyocytes induced by OGDR. Our finding of reduced level of the CRAMP peptide in I/R heart tissues is contradictory to a previous report, which showed increased CRAMP mRNA expression in murine ventricular tissues after I/R injury using a Langendorff system [34]. This may be due to the fact that in our study we used an in vivo I/R model, whereas Karapetyan et al. utilized an ex vivo I/R model. Furthermore, the reperfusion time was 24 h in our study compared to only 20 min for reperfusion in the reported study [34]. In addition to the reduced level of the CRAMP peptide in apoptotic cardiomyocytes, we also observed decreased serum level of the mCRAMP peptide in the murine model of I/R injury, as well as reduced serum level of the human cathelicidin LL-37 in MI patients compared to healthy subjects. These results consistently suggest a potential role of CRAMP in cardiomyocyte apoptosis and ischemic myocardial injury. Notably, in OGDR-treated cardiomyocytes, treatment with the CRAMP peptide reduced apoptosis, whereas knockdown of CRAMP enhanced apoptosis. Furthermore, intraperitoneal injections of the CRAMP peptide were able to reduce infarct size and attenuate myocardial apoptosis in I/R mice, whereas CRAMP knockout mice displayed aggravated I/R injury. In addition to reduced infarct size and myocardial apoptosis, cardiac function after I/R injury deserves further investigation, which is a limitation of the present study. Moreover, although we detected a predominant expression of CRAMP in cardiomyocytes compared to fibroblasts, it also deserves investigating the expression of CRAMP in other types of cells such as endothelial cells and immune cells upon cardiac I/R injury. Collectively, we provide direct evidence that CRAMP is protective against I/R injury and cardiomyocyte apoptosis.

The activation of Akt and ERK1/2 pathways confer cardioprotection in many settings including I/R injury [19, 23, 26, 35–37]. Here, we demonstrated that the CRAMP peptide induced Akt and ERK1/2 phosphorylation in cardiomyocytes at baseline and in OGDR-treated cardiomyocytes, whereas knockdown of CRAMP had opposite effects. Notably, the protective effect of the CRAMP peptide in reducing OGDR-induced apoptosis was abolished by the Akt inhibitor MK2206 and the MEK inhibitor PD98059. These results reveal that the CRAMP peptide reduces cardiomyocyte apoptosis at least in part via the activation of Akt and ERK1/2 pathways. Here, one limitation is that the Akt and MEK inhibitors were not given in vivo in the presence of the mCRAMP peptide treatment in the I/R mouse model. The function-rescue in vivo study will be of great interest to define the contribution of Akt and ERK1/2 in mediating the protective effect of CRAMP against cardiac I/R injury.

Several studies have reported that Akt and ERK1/2 activation can further lead to FoxO3a phosphorylation and export from the nucleus [38–40], thus reducing ischemic myocardial injury by inhibiting cellular apoptosis [28]. FoxO3a belongs to the forkhead family of transcriptional regulators [40–42]. Nuclear FoxO3a induces transcription of pro-apoptotic genes including Bim [43], whereas FoxO3a phosphorylation and export from the nucleus leads to reduced transcription of Bim, further reducing pro-apoptotic protein Bax and increasing anti-apoptotic protein Bcl-2 [28]. In the present study, we demonstrated that OGDR treatment reduced phosphorylation level of FoxO3a and increased nuclear accumulation of FoxO3a in cardiomyocytes, and knockdown of CRAMP further enhanced these phenomena. In contrast, treatment with the CRAMP peptide led to increased FoxO3a phosphorylation and nuclear export in OGDR-treated cardiomyocytes. Moreover, we observed that the reduced phosphorylation levels of Akt, ERK1/2, and FoxO3a in the murine model of I/R injury could be reversed by treatment with the CRAMP peptide, whereas CRAMP knockout mice exhibited further reduced phosphorylation levels of Akt, ERK1/2, and FoxO3a compared to wild type mice in the condition of I/R injury. Thus, our results support that the protective effect of CRAMP against cardiomyocyte apoptosis is closely related to Akt and ERK1/2 activation and FoxO3a phosphorylation and nuclear export. Actually, the phosphorylation of FoxO3a has been reported to be altered either at S253 or at T32 (or not clearly indicated), depending on different models and disease stages of ischemic or hypoxic cardiac injury [28, 44–46]. In the present study, we found that the phosphorylation of FoxO3a was reduced at S253 but not T32 in OGDR-treated NRCMs as well as in the heart upon I/R injury. Moreover, the CRAMP peptide led to increased FoxO3a phosphorylation at S253, while reducing CRAMP had opposite effect. The results of FoxO3a phosphorylation in the present study were consistent in vitro and in vivo; however, it still deserves investigating the mechanism responsible for the phosphorylation of FoxO3a at different sites.

c-Jun belongs to the Jun family and is an important component of the transcription factor activator protein-1 (AP-1), which plays crucial roles in a variety of biological processes including cell proliferation, differentiation, and apoptosis [47–51]. Increased c-Jun transcription and activity induced by activation of c-Jun NH_2_-terminal kinase (JNK) has been implicated in the pathogenesis of myocardial I/R injury and cardiomyocyte apoptosis [52–57]. In contrast, overexpression of c-Jun or activation of JNK may also have anti-apoptotic effects under certain circumstances [58, 59]. JNK activation was reported to promote cardiomyocyte survival after oxidative stress, and the protective effect may be related to reactivation of Akt [60, 61]. Thus, the roles of c-Jun in cell apoptosis are multi-facetted, which are cell type and condition dependent [62]. Using the Genomatix Software Suite database, we identified c-Jun as a potential upstream regulator of CRAMP and further validated that knockdown of c-Jun upregulated the CRAMP mRNA level in cardiomyocytes. Moreover, we showed that c-Jun was increased in apoptotic cardiomyocytes induced by OGDR and in heart tissues from I/R mice, and found that knockdown of c-Jun could inhibit OGDR-induced cardiomyocyte apoptosis, whereas this effect was totally attenuated by CRAMP siRNA. Thus, our results identify c-Jun as a negative regulator of CRAMP and confirm that inhibition of c-Jun is protective against OGDR-induced cardiomyocyte apoptosis.

Increasing evidence has shown that circulating antimicrobial peptide levels can change upon different stress or injury including infection, inflammation, atherosclerosis, and MI [11]. Plasma defensin levels were markedly increased in patients with septicemia or bacterial meningitis most likely reflecting neutrophil activation [63]. The deposition of α-defensin in the skin was demonstrated to be an independent predictor for coronary artery disease severity, linking inflammation to atherosclerosis [31]. In a prospective study of a type I diabetic cohort, increased plasma level of α-defensin was proved to be associated with increased cardiovascular morbidity and mortality [64]. In addition to increased level of α-defensin, a recent study showed reduced level of LL-37 in the plasma from patients with acute MI [30]. However, there is still a lack of data regarding the clinical relevance of circulating LL-37 in the prognosis of MI. In our study, we observed reduced LL-37 serum level in MI patients, with a much lower level in MI patients with readmission and/or death compared to those patients that survived during the 1-year follow-up. These results suggest a potential value of circulating LL-37 as a risk marker for worse prognosis in MI patients. Logistic regression further confirmed that serum level of LL-37 was not associated with other clinical characteristics of MI patients and indicated that serum LL-37/neutrophil ratio inversely correlated with readmission and/or death in MI patients. Future large-scale and multicenter prospective studies will be needed to confirm the prognostic value of serum LL-37/neutrophil ratio as a biomarker for readmission and/or death in patients with MI.

## Conclusions

We demonstrate that CRAMP is protective against myocardial apoptosis upon I/R injury. The activation of Akt and ERK1/2 pathways and the phosphorylation and nuclear export of FoxO3a contribute to the cardioprotective effect of CRAMP. We also show that lower serum LL-37/neutrophil ratio may be a potential predictor for worse prognosis of MI patients. These findings improve our understanding of the roles of antimicrobial peptides in cardiovascular diseases and suggest that increasing the level of the human cathelicidin LL-37 might be a novel therapeutic strategy for cardiac ischemic injury.

## Additional files

Additional file 1: **Figure S1.** Western blot analysis for Akt and ERK1/2 phosphorylation level in neonatal rat cardiomyocytes (NRCMs) treated with Akt or MEK inhibitor. (a) NRCMs were treated with Akt inhibitor MK2206 (10 nM, 24 h) or control under the condition of oxygen glucose deprivation/reperfusion (OGDR) (*n* = 3). (b) NRCMs were treated with MEK inhibitor PD98059 (50 μM, 24 h) or control under the condition of OGDR (*n* = 3). All membranes were probed, stripped, and then reprobed for determining the phosphorylation levels of Akt and ERK1/2. Data were expressed as mean ± SD. *, *P* < 0.05; **, *P* < 0.01; ***, *P* < 0.001. (PDF 148 kb)

Additional file 2: **Figure S2.** Intraperitoneal injection of the mCRAMP peptide increases the level of mCRAMP in both serum and heart samples of mice with ischemia-reperfusion (I/R) injury. (a) The level of the mCRAMP peptide was measured by ELISA in the serum from I/R mice (*n* = 7). (b-c) The level of the mCRAMP peptide was measured by ELISA in the infarct (b) and border (c) zones from I/R hearts (*n* = 5). Data were expressed as mean ± SD. *, *P* < 0.05; **, *P* < 0.01; ***, *P* < 0.001. (PDF 62 kb)

Additional file 3: **Figure S3.** Western blot analysis for the Bax/Bcl ratio, Caspase-3 cleavage, and phosphorylation levels of Akt, ERK1/2, and FoxO3a in mouse ischemia/reperfusion (I/R) hearts. The Bax/Bcl ratio and Caspase-3 cleavage, as well as the Akt, ERK1/2, and FoxO3a phosphorylation levels, were analyzed by Western blot in the infarct, border, and remote zones of mouse I/R hearts (*n* = 3). All membranes were probed, stripped, and then reprobed for determining the phosphorylation levels of Akt, ERK1/2, and FoxO3a. Data were expressed as mean ± SD. *, *P* < 0.05; **, *P* < 0.01; ***, *P* < 0.001. (PDF 166 kb)

Additional file 4: **Figure S4.** The cardiac phenotypes of mCRAMP knockout mice at baseline. (a) The heart weight, body weight, tibia length, as well as the heart weight/body weight ratio and the heart weight/tibia length ratio were measured in mCRAMP knockout mice and WT mice (*n* = 5). (b) Representative images of Hematoxylin-Eosin staining for heart tissues. Scale bar = 50 μm. (c) The wheat germ agglutinin (WGA) staining for myocardial cross-sectional area (*n* = 5). Scale bar = 25 μm. (d) qRT-PCRs for ANP and BNP expression levels in heart tissues from mCRAMP knockout mice and WT mice (*n* = 6). Data were expressed as mean ± SD. (PDF 282 kb)

Additional file 5: **Figure S5.** qRT-PCR for c-Jun and Rela in neonatal rat cardiomyocytes transfected with their respective siRNAs. The mRNA levels of c-Jun and Rela were measured by qRT-PCR in neonatal rat cardiomyocytes transfected with siRNAs targeting c-Jun and Rela (*n* = 6), c-Jun-siRNA-2 and Rela-siRNA-1 were then used in functional experiments. Data were expressed as mean ± SD. ***, *P* < 0.001. (PDF 74 kb)
