# Supplementary figures and images for: Cathelicidin-related antimicrobial peptide protects against myocardial ischemia/reperfusion injury
Source: BMC Med. 2019 Feb 20;17:42. doi: 10.1186/s12916-019-1268-y (PMC6381635; doi:10.1186/s12916-019-1268-y)

# Supplementary Figure 1

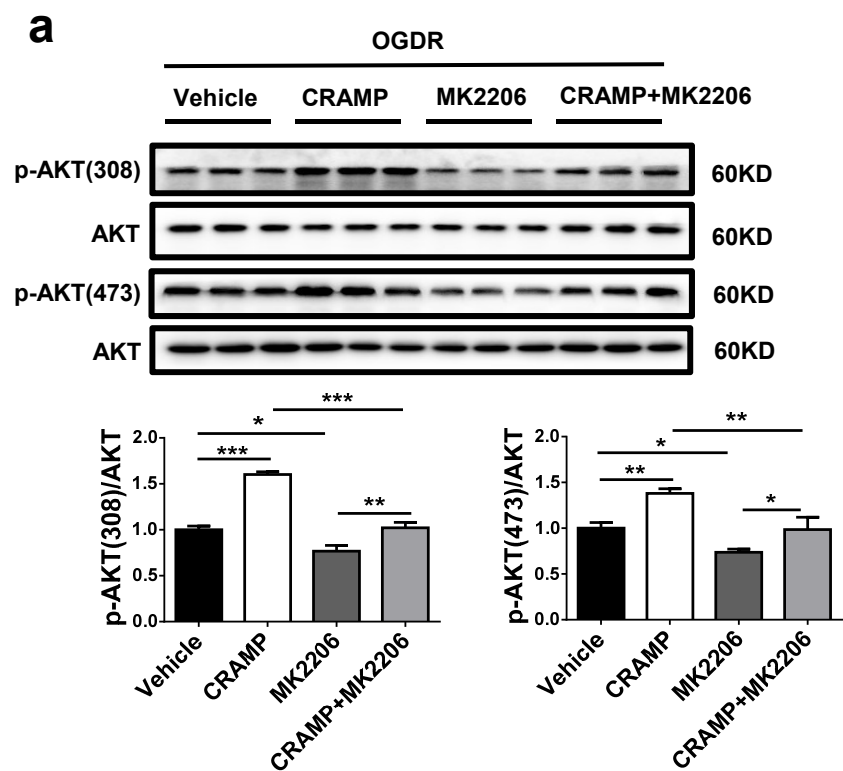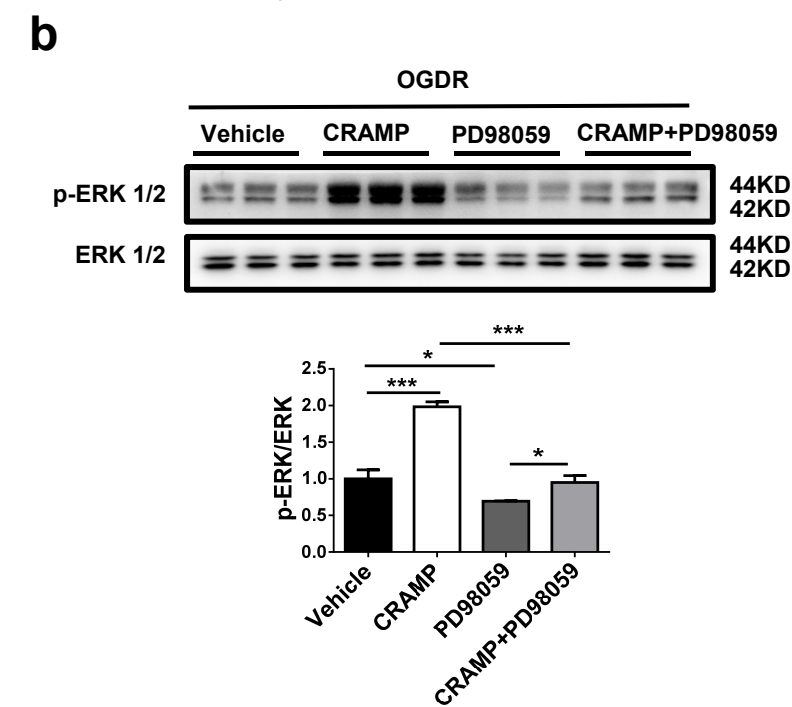

Supplement: Supplementary file 1 — Figure S1. Western blot analysis for Akt and ERK1/2 phosphorylation level in neonatal rat cardiomyocytes (NRCMs) treated with Akt or MEK inhibitor. (a) NRCMs were treated with Akt inhibitor MK2206 (10 nM, 24 h) or control under the condition of oxygen glucose deprivation/reperfusion (OGDR) (n = 3). (b) NRCMs were treated with MEK inhibitor PD98059 (50 μM, 24 h) or control under the condition of OGDR (n = 3). All membranes were probed, stripped, and then reprobed for determining the phosphorylation levels of Akt and ERK1/2. Data were expressed as mean ± SD. *, P < 0.05; **, P < 0.01; ***, P < 0.001. (PDF 148 kb) [file 12916_2019_1268_MOESM1_ESM.pdf]

# Supplementary Figure 2

a

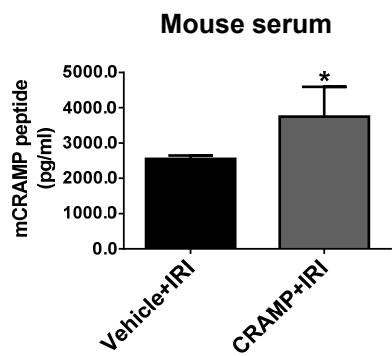

b

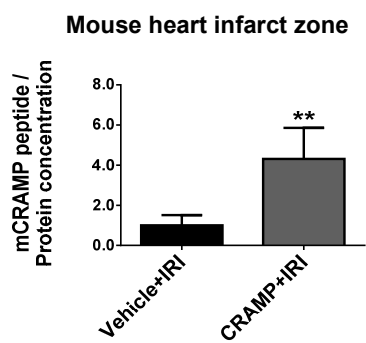

c

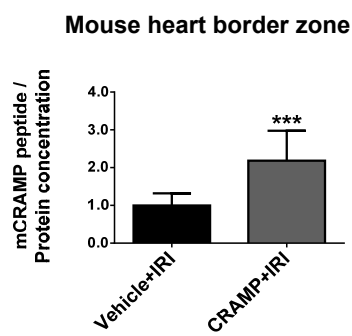

Supplement: Supplementary file 2 — Figure S2. Intraperitoneal injection of the mCRAMP peptide increases the level of mCRAMP in both serum and heart samples of mice with ischemia-reperfusion (I/R) injury. (a) The level of the mCRAMP peptide was measured by ELISA in the serum from I/R mice (n = 7). (b-c) The level of the mCRAMP peptide was measured by ELISA in the infarct (b) and border (c) zones from I/R hearts (n = 5). Data were expressed as mean ± SD. *, P < 0.05; **, P < 0.01; ***, P < 0.001. (PDF 62 kb) [file 12916_2019_1268_MOESM2_ESM.pdf]

# Supplementary Figure 3

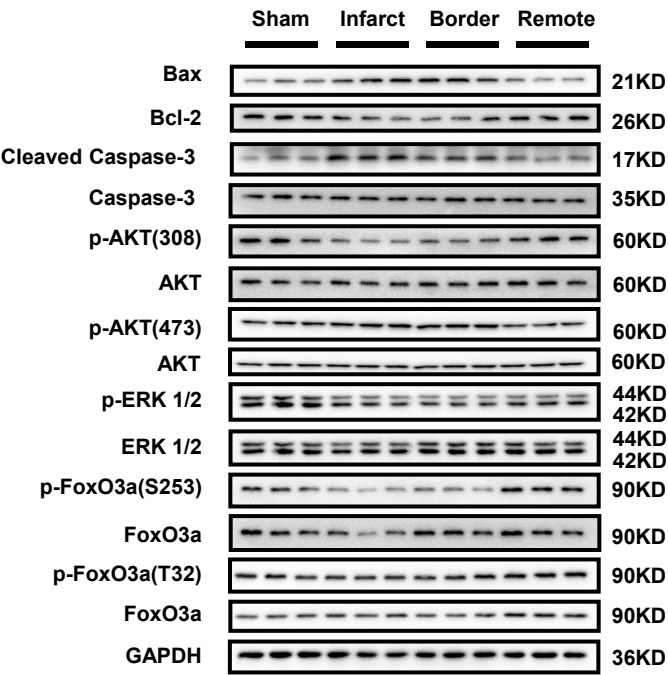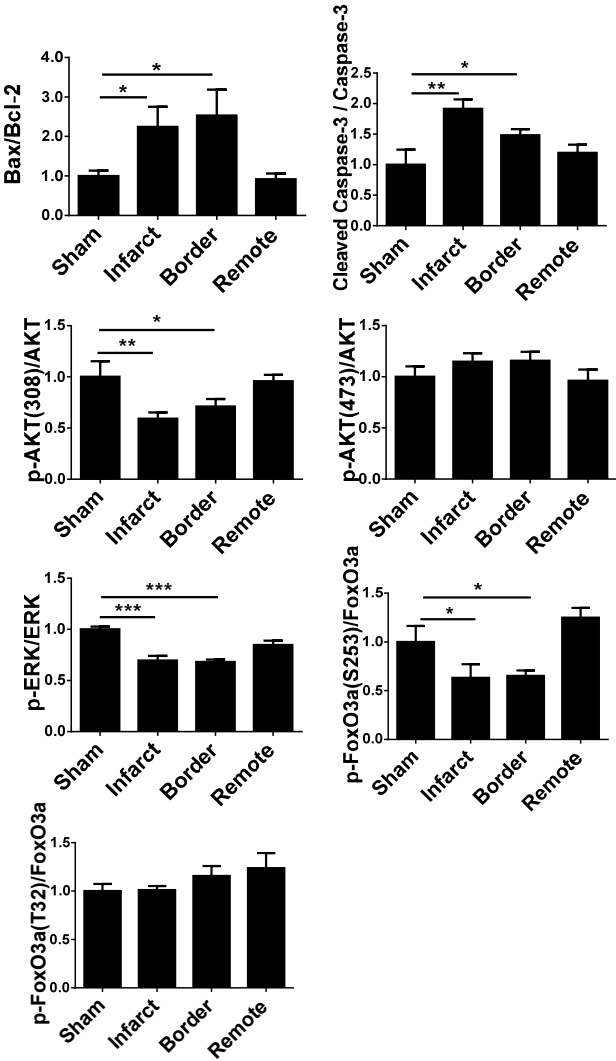

Supplement: Supplementary file 3 — Figure S3. Western blot analysis for the Bax/Bcl ratio, Caspase-3 cleavage, and phosphorylation levels of Akt, ERK1/2, and FoxO3a in mouse ischemia/reperfusion (I/R) hearts. The Bax/Bcl ratio and Caspase-3 cleavage, as well as the Akt, ERK1/2, and FoxO3a phosphorylation levels, were analyzed by Western blot in the infarct, border, and remote zones of mouse I/R hearts (n = 3). All membranes were probed, stripped, and then reprobed for determining the phosphorylation levels of Akt, ERK1/2, and FoxO3a. Data were expressed as mean ± SD. *, P < 0.05; **, P < 0.01; ***, P < 0.001. (PDF 166 kb) [file 12916_2019_1268_MOESM3_ESM.pdf]

# Supplementary Figure 4

a

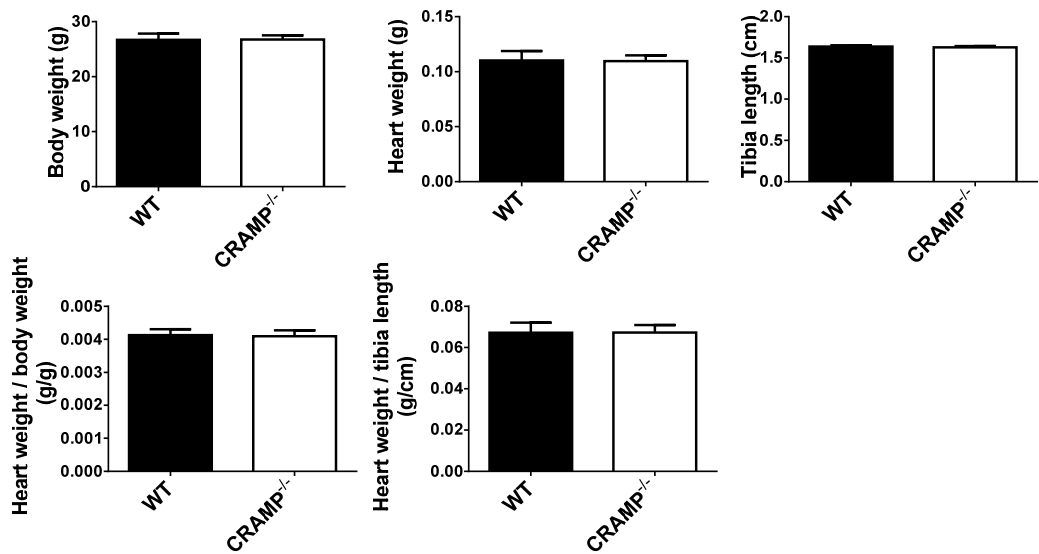

b

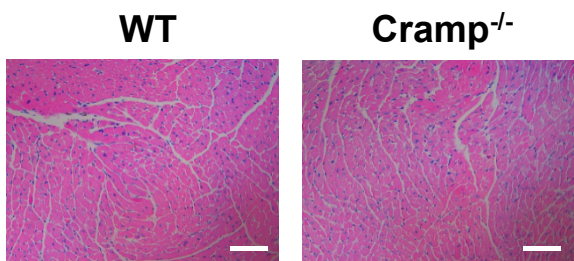

c

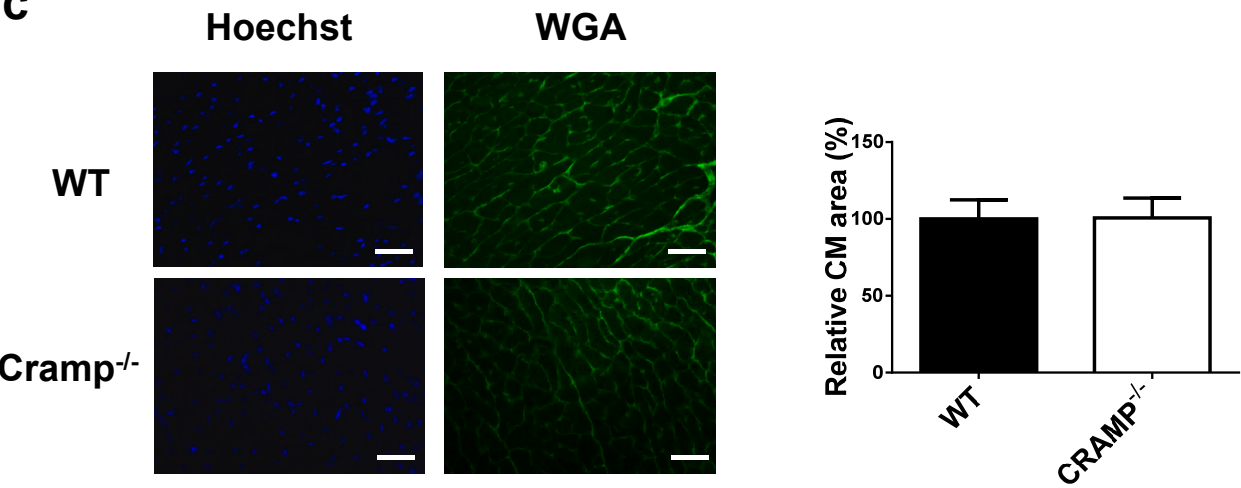

d

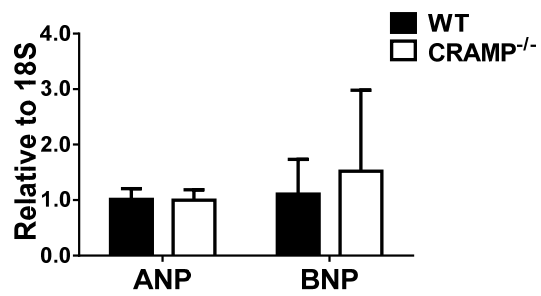

Supplement: Supplementary file 4 — Figure S4. The cardiac phenotypes of mCRAMP knockout mice at baseline. (a) The heart weight, body weight, tibia length, as well as the heart weight/body weight ratio and the heart weight/tibia length ratio were measured in mCRAMP knockout mice and WT mice (n = 5). (b) Representative images of Hematoxylin-Eosin staining for heart tissues. Scale bar = 50 μm. (c) The wheat germ agglutinin (WGA) staining for myocardial cross-sectional area (n = 5). Scale bar = 25 μm. (d) qRT-PCRs for ANP and BNP expression levels in heart tissues from mCRAMP knockout mice and WT mice (n = 6). Data were expressed as mean ± SD. (PDF 282 kb) [file 12916_2019_1268_MOESM4_ESM.pdf]

Supplementary Figure 5

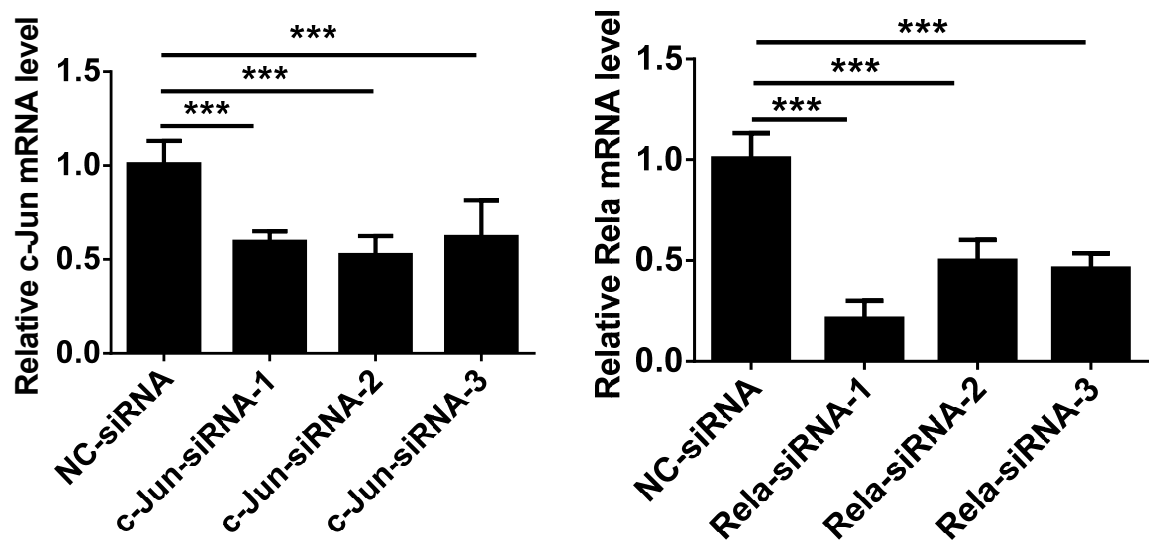

Supplement: Supplementary file 5 — Figure S5. qRT-PCR for c-Jun and Rela in neonatal rat cardiomyocytes transfected with their respective siRNAs. The mRNA levels of c-Jun and Rela were measured by qRT-PCR in neonatal rat cardiomyocytes transfected with siRNAs targeting c-Jun and Rela (n = 6), c-Jun-siRNA-2 and Rela-siRNA-1 were then used in functional experiments. Data were expressed as mean ± SD. ***, P < 0.001. (PDF 74 kb) [file 12916_2019_1268_MOESM5_ESM.pdf]
